# Intravenous galanin (1–16) and a GalR1 agonist attenuate allodynia in rats with spared nerve injury

**DOI:** 10.1097/PR9.0000000000001422

**Published:** 2026-03-10

**Authors:** Bernardo Miguel, Marcelo J. Villar, Tobías Giovannetti, Valentino Desanto, Sofía Villalba, Verónica Bisagno, Mailín Casadei, Tambet Teesalu, Tomas Hökfelt, Pablo R. Brumovsky

**Affiliations:** a Instituto de Investigaciones en Medicina Traslacional CONICET-Universidad Austral, Buenos Aires, Argentina; b Facultad de Ciencias Biomédicas, Universidad Austral, Buenos Aires, Argentina; c Faculty of Biomedical Sciences, University of Tartu, Tartu, Estonia; d Department of Neuroscience, Karolinska Institutet, Stockholm, Sweden

**Keywords:** Blood-brain barrier, GalR1 receptor, Neuropathic pain, Neuropeptide, Mechanical allodynia

## Abstract

**Introduction:**

Chronic pain is a major unmet medical need, with current treatments often providing limited relief. The neuropeptide galanin, acting through GalR1-3 receptors, has been implicated in pain modulation. Although galanin analogues are typically administered intrathecally, emerging evidence suggests that peptides can cross the blood–brain barrier. We hypothesized that systemic administration of the biologically active fragment galanin (1–16) could attenuate neuropathic pain.

**Objectives:**

To evaluate whether intravenous galanin (1–16) reduces pain-like behaviors in a rat model of neuropathic pain and to compare its effects when using a selective GalR1 agonist.

**Methods:**

Male Sprague–Dawley rats underwent spared nerve injury. Animals received intravenous galanin (1–16), M617 (selective GalR1), or vehicle. Mechanical and cold allodynia were assessed using von Frey filaments and acetone testing, respectively. Open-field tests evaluated locomotor activity to exclude more general behavioral effects. Data were analyzed using blinded protocols and appropriate statistical tests.

**Results:**

Galanin (1–16) and M617 significantly reversed mechanical allodynia compared with vehicle, with effects evident at 0.5 hours, peaking at 2 hours, still present after 4 hours, and absent by 6 hours (*P* < 0.0001). No significant differences were observed between galanin (1–16) and M617. By contrast, neither compound significantly affected cold allodynia. Locomotor activity, total distance travelled, or center time were not affected by galanin (1–16).

**Conclusion:**

Intravenous administration of galanin (1–16) produces robust but transient antiallodynic effects in neuropathic rats, likely mediated through GalR1 activation, without impairing locomotion. These findings highlight the therapeutic potential of systemically administered galanin or galanin analogues targeting GalR1 for neuropathic pain management.

## Abstract 2

Intravenous galanin (1–16) alleviates mechanical allodynia in neuropathic rats, likely via GalR1 activation, without locomotor effects, supporting its potential as a systemic analgesic strategy

## 1. Introduction

Chronic pain is a serious and debilitating neurological condition affecting around 25% of adults aged 25+ years worldwide.^42^ Currently, there is an urgent need for novel strategies for treatment of this type of pain. Administration of opioid analgesics^36^ as well as antiepileptic drugs or antidepressants^1^ often have, for various reasons, disappointing therapeutic outcomes. Likewise, major efforts in academia and pharmaceutical companies have not led to a breakthrough.

The neuropeptide galanin,^32^ acting through 3 G protein–coupled receptors, GalR1-3,^20^ can be detected at the spinal level both in the dorsal horn and, at low levels, in cell bodies in dorsal root ganglia (DRGs).^6,8,19,31^ However, after peripheral axotomy, there is a dramatic increase in galanin levels in the DRG neuronal cell bodies.^33^ This finding raised interest in a possible role of galanin in pain signaling,^40^ and the concept that galanin has both pro- and antinociceptive effects after nerve injury has emerged.^21^ Thus, galanin, at low intrathecal doses and initially after injury, exerts a pronociceptive effect, whereas at later phases and larger doses, it relieves pain, likely mediated by GalR1 receptors on interneurons in the spinal dorsal horn.^17,39^ These findings highlight the potential of GalR1 agonists for the treatment of neuropathic pain. In fact, such a selective peptide GalR1 agonist has been generated, termed M617.^23^

In our own extensive experiments, the intrathecal route for administration of galanin/galanin analogues was used because we assumed that peptides do not pass the blood–brain barrier (BBB). However, increasing evidence indicates that peptides indeed can penetrate the BBB and exert distinct central effects,^2^ a remarkable example being the glucagon-like peptide-1 agonists for treatment of, initially, diabetes type 2.^25^ In the present study, we tested the hypothesis that peripheral administration of the fragment galanin (1–16) may cause pain relief in rats with neuropathic pain. This fragment has the full biological activity of galanin (1–29).^12^ We studied the effect on responses to mechanical and temperature stimuli. For comparison, we tested M617. Finally, we evaluated patterns of locomotor activity to exclude more general behavioral effects.

## 2. Methods

### 2.1. Animals

Sixty-four Sprague−Dawley male rats (200−250 g, 6 weeks old; Facultad de Farmacia y Bioquímica, Universidad de Buenos Aires) were used. Animals were housed under controlled temperature and a 12-hour-light/dark cycle, with food and water ad libitum. A 1-week housing habituation preceded experiments. All procedures were approved by the Institutional Animal Care and Use Committee (2024-09) at Instituto de Investigaciones en Medicina Traslacional and followed National Institutes of Health guidelines (Publication 86-23).

### 2.2. Experimental drugs

Galanin (1–16) and M617 (Chemtronica, Sollentuna, Sweden) were prepared as 4 mM working solutions in sterile phosphate-buffered saline (PBS) 1X with 2% dimethyl sulfoxide (DMSO). Compounds were administered through the tail vein in a body weight–adjusted volume to achieve a 100-µM estimated blood peak concentration (0.45–0.55 mL). Sterile PBS 1X with 2% DMSO served as vehicle control.

### 2.3. The spared nerve injury model

The spared nerve injury (SNI) model, involving tight ligation and distal axotomy of the common peroneal and tibial branches of the sciatic nerve, was performed as described by Decosterd et al.^11^

### 2.4. Behavioral assessment

**Figure 1.**
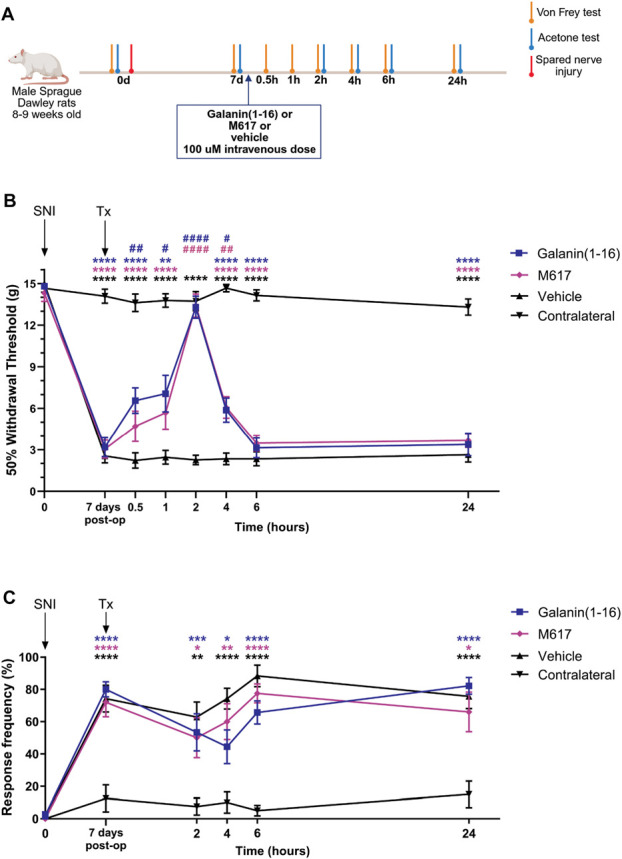
Schematic drawing depicting the experimental design of pain-like behavior tests in this study (A), as well as mechanical (B) and cold (C) allodynia assessment in rats with SNI, before (baseline) and after IV administration of galanin (1–16) (n = 9), M617 (n = 10), or vehicle (n = 13). Statistical significance was established by means of 2-way ANOVA followed by the Bonferroni post hoc test: # or *P < 0.05; ## or **P < 0.01; ### or ***P < 0.001; #### or ****P < 0.0001. VEH vs galanin (1–16) (# in blue) or VEH vs M617 (# in violet); contralateral vs galanin (1–16) (* in blue) or contralateral vs M617 (* in violet) or contralateral vs VEH (* in black). Of note, Bonferroni test showed the following significant differences: at 0.5 hours after treatment, galanin (1–16) vs vehicle (6.56 ± 0.91 vs 2.22 ± 0.55, *P* = 0.0037); at 1 hour after treatment, galanin (1–16) vs vehicle (7.06 ± 1.31 vs 2.45 ± 0.55, *P* = 0.0240); at 2 hours after treatment, galanin (1–16) vs vehicle (13.293 ± 0.79 vs 2.26 ± 0.34, *P* < 0.0001) and M617 vs vehicle (13.40 ± 0.83 vs 2.26 ± 0.34, *P* < 0.0001); 4 hours after treatment, galanin (1–16) vs vehicle (5.87 ± 0.87 vs 2.34 ± 0.42, *P* = 0.0108) and M617 vs vehicle (6.07 ± 0.77 vs 2.34 ± 0.42, *P* = 0.0026). INJ, IV drug injection; SNI, spared nerve injury; post-op, postoperative; VEH, vehicle.

Behavioral testing was conducted during daytime (9.00−18.00 hours). After 30 minutes of habituation, mechanical and cold sensitivity of the lateral plantar surface of both ipsilateral and contralateral hind paws was assessed (see below). Because both modalities were evaluated in the same animals, mechanical thresholds were measured at 0.5, 1, 2, 4, 6, and 24 hours postadministration, whereas cold allodynia was measured only at 2, 4, 6, and 24 hours (30 minutes after each mechanical allodynia testing). Early cold testing was omitted due to insufficient intervals between mechanical assessments (Fig. 1A).

Open-field testing was performed between 9.00 and 13.00 hours, in a sound-attenuated room using a nonreflective black plastic arena (68 × 68 × 45 cm).

#### 2.4.1. Mechanical and cold allodynia

Mechanical sensitivity was measured by use of 0.6, 1.4, 2, 4, 6, 8, 10, and 15 von Frey nylon monofilaments (Stoelting Inc, Wooddale, IL) and the modified up–down method of Dixon, as described by Chaplan et al.,^9^ to determine the 50% withdrawal threshold. Cold allodynia was evaluated using the Choi acetone test^10^ and expressed as the percentage of positive withdrawals across 5 stimulations. Testing was performed at baseline (24 hours before surgery) and 7 days postsurgery. Subsequently, rats received intravenous galanin (1–16), M617, or vehicle and were tested at the indicated time points (Fig. 1A).

Animals failing to develop mechanical allodynia (threshold of ≤6 g at day 7) were excluded (n = 6).

#### 2.4.2. Locomotion assessment

Locomotor activity was recorded simultaneously in 2 open-field arenas using Ethovision XT 7.0 software (Noldus, Wageningen, The Netherlands).^28^ Spared nerve injury rats were assigned to vehicle or galanin (1–16) groups. Two hours after drug administration, animals were placed in the arena center and recorded for 1 hour. Distance traveled was analyzed in twelve 5-minute bins or as a total distance. Time spent in the center (T_center_) was calculated as the summed duration within the central zones (∑time_centralzones_).

### 2.5. Data analysis

Experiments were conducted blinded. Data are presented as mean ± SEM and analyzed using GraphPad Prism 8. Mechanical and cold allodynia were analyzed by 2-way ANOVA followed by Bonferroni post hoc tests (contralateral paw responses did not differ between the groups and were pooled). Open field data were analyzed using unpaired *t* test or 2-way ANOVA for time–bin comparisons. Statistical significance was set at *P* < 0.05.

## 3. Results

All rats included in this analysis exhibited significant ipsilateral mechanical and cold allodynia by day 7 after SNI, as compared with baseline and contralateral hind paws; rats receiving vehicle remained allodynic during the entire tested period (Figs. 1B and C).

Analysis of mechanical allodynia revealed that intravenous galanin (1–16) and M617 significantly reduce pain-like behavior, as reflected by an increase in mechanical thresholds (2-way ANOVA: <0.0001). For both agonists, the effect appeared within the first hour, followed by a peak after 2 hours, and a quick decline in mechanical thresholds, albeit still significantly different as compared with vehicle at 4 hours postadministration (Fig. 1B). Mechanical allodynia in all animals returned to baseline by 6 hours after treatment. No differences were detected in the antiallodynic effects between galanin (1–16) and M617 (Fig. 1B).

**Figure 2.**
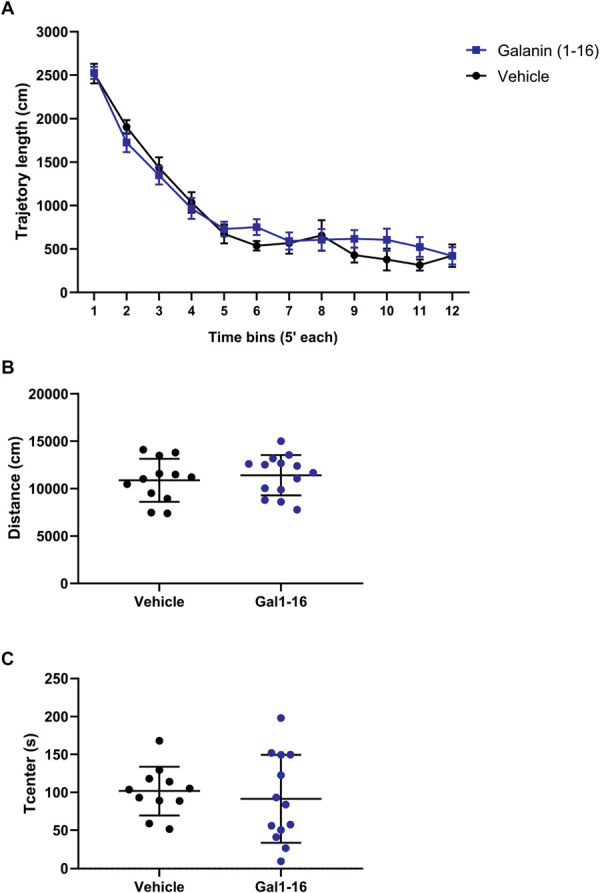
Locomotion assessment in rats with 7-day SNI, receiving either galanin (1–16; n = 12) or vehicle (n = 14). Distance through 12 five-minute time bins (A), total distance travelled throughout the session (B), and time spent in the center of the arena (T_center_) were assessed (C). Data are presented as mean ± SEM. Statistical comparisons between the groups were performed using unpaired *t* test or 2-way ANOVA when analyzing time bins. No significant differences were detected (*P* > 0.05). SNI, spared nerve injury.

By contrast, analysis of cold allodynia did not reveal any major antiallodynic effect for either compound (Fig. 1C). Finally, analysis of locomotor activity in rats receiving either galanin (1–16) or vehicle revealed similar locomotive habituation in both groups throughout the test (Fig. 2A), and no significant differences in total distance travelled (Fig. 2B) or in T_center_ values (Fig. 2C).

## 4. Discussion

Most foundational work on galanin-mediated pain modulation over the past 3 decades has relied on intrathecal administration of peptide ligands, based on the assumption that peptides do not cross the BBB. Consequently, significant medicinal chemistry efforts have focused on improving the poor pharmacokinetic properties of native galanin, leading to the development of galanin-mimetic, nonpeptidergic analogues such as galnon and galmic.^3,4,30,37^ These ligands produce antinociceptive effects in rodent pain models^4,37^ but interact with multiple G Protein-Coupled Receptor (GPCRs) and lack strict galanin-receptor selectivity.^22^ In parallel, high-affinity, systemically active galanin peptide analogues have been developed, showing anticonvulsant effects after intraperitoneal administration—an activity not observed with galanin (1–16).^7^ Further optimization yielded analogues with antinociceptive activity after intraperitoneal, subcutaneous, or oral administration in the intraplantar formalin model, although effects in neuropathic pain were not examined.^29^

Here, we demonstrate that intravenous galanin (1–16) produces a transient but robust blockade of mechanical allodynia in rats with neuropathic pain, an effect likely involving GalR1 activation. These findings complement clinical evidence supporting systemic galanin activity in humans, including rapid antidepressant effects in patients with depression^27^ and increased duration of the third rapid eye movement (REM) sleep cycle in healthy young volunteers.^26^ However, galanin is a neurohormone,^41^ with documented effects on plasma glucose regulation (presumably via GalR1),^24^ growth hormone release,^34^ and postprandial gastrointestinal motility.^5^ Thus, development of galanin-based analgesics, particularly when chronic treatments are needed, will require strategies that preserve antinociceptive efficacy while minimizing systemic side effects.

A key unresolved issue is whether the antiallodynic effects of intravenous galanin (1–16) or M617 are mediated centrally, peripherally, or both. Extensive evidence that intrathecal galanin, galanin analogues, and selective GalR1 agonists produce *central* antiallodynic effects^15,20,35,38,40^ supports the possibility of BBB penetration. *Peripheral* galaninergic actions are more complex, encompassing both pro- and antinociceptive effects. Thus, peripheral galanin exposure reveals 2 afferent nerve populations, with a larger one showing inhibition and a smaller subset exhibiting sensitization.^13^ Consistent with this, intraplantar M617 has been shown to be antinociceptive in naive or capsaicin-sensitized rats, whereas the GalR2-preferring agonist AR-M1896 exhibited pronociceptive actions.^18^ In addition, intradermal galanin or Gal (2–11) (a selective GalR2/3 agonist) modulates mechanosensitive C-fibers in a concentration-dependent manner, sensitizing nociceptors at low doses and inhibiting them at higher doses.^16^ Notably, intra-arterial galanin administered near the knee joint, at concentrations comparable to those used here, reduces mechanosensitivity in primary afferent terminals.^14^ Together, these findings support GalR1 as central and potentially peripheral mediator of the antiallodynic effects of galanin (1–16).

## Disclosures

The authors have no conflict of interest to declare.
